# Hypoxia-Inducible Factor Regulates Endothelial Metabolism in Cardiovascular Disease

**DOI:** 10.3389/fphys.2021.670653

**Published:** 2021-07-05

**Authors:** Karim Ullah, Rongxue Wu

**Affiliations:** ^1^Biochemistry and Molecular Medicine, University of Oulu, Oulu, Finland; ^2^Biological Sciences Division, Department of Medicine, University of Chicago, Chicago, IL, United States

**Keywords:** hypoxia-inducible factor, endothelial metabolism, cardiovascular disease, myocardial ischemia, atherosclerosis, pulmonary hypertension, diabetic endothelial dysfunction

## Abstract

Endothelial cells (ECs) form a physical barrier between the lumens and vascular walls of arteries, veins, capillaries, and lymph vessels; thus, they regulate the extravasation of nutrients and oxygen from the circulation into the perivascular space and participate in mechanisms that maintain cardiovascular homeostasis and promote tissue growth and repair. Notably, their role in tissue repair is facilitated, at least in part, by their dependence on glycolysis for energy production, which enables them to resist hypoxic damage and promote angiogenesis in ischemic regions. ECs are also equipped with a network of oxygen-sensitive molecules that collectively activate the response to hypoxic injury, and the master regulators of the hypoxia response pathway are hypoxia-inducible factors (HIFs). HIFs reinforce the glycolytic dependence of ECs under hypoxic conditions, but whether HIF activity attenuates or exacerbates the progression and severity of cardiovascular dysfunction varies depending on the disease setting. This review summarizes how HIF regulates the metabolic and angiogenic activity of ECs under both normal and hypoxic conditions and in a variety of diseases that are associated with cardiovascular complications.

## Introduction

Eukaryotic cells produce adenosine triphosphate (ATP) primarily via two linked processes: glycolysis, which occurs in the cytosol, and oxidative phosphorylation (OXPHOS), which uses one of the products of glycolysis and pyruvate and occurs in the mitochondria. Of the two, OXPHOS is much more efficient (Falkenberg et al., 2019), producing 12–14-fold more ATP per molecule of substrate, and numerous pathophysiological conditions (e.g., anemia, myocardial infarction, inflammation) (Lopez-Barneo et al., 2001; Cornet et al., 2013) can occur under hypoxic conditions when the oxygen supply fails to meet the demand. Hypoxia also contributes to normal embryonic development, wound healing, and cell proliferation (Semenza, 2003; Fathollahipour et al., 2018), but when caused by an abnormal decline in oxygen tension (Stamati et al., 2011) or an interruption in blood flow (ischemia), it activates a network of protective mechanisms that can be collectively called the hypoxia response pathway.

The master regulators of the hypoxia response pathway are hypoxia-inducible factors (HIFs), and consequently, HIFs control a broad range of mechanisms that have key roles in the growth, differentiation, survival, and metabolic activity of cells, as well as embryonic development, angiogenesis, and numerous other physiological processes (Schofield and Ratcliffe, 2004; Semenza, 2014). HIFs also influence the expression of numerous molecules that regulate the survival, metabolism, and angiogenic activity of vascular endothelial cells (ECs) (Fraisl et al., 2009), which serve not only as a physical barrier between the vessel wall and the lumen but also secrete numerous factors that have key roles in maintaining cardiovascular health. Thus, endothelial dysfunction can be a major contributor to inflammation (Sun et al., 2019), as the immune system responds to the extravasation of molecules from the circulation, to the cardiovascular complications associated with many diseases or physiological conditions (Maltepe et al., 1997; Hink et al., 2001; Falkenberg et al., 2019), and to the progression of diseases such as cancer, which are driven by aberrations in vessel growth (Vasa et al., 2001; Matsuzawa and Lerman, 2014; Falkenberg et al., 2019). In this review, we briefly summarize how HIF regulates the metabolic and angiogenic activity of ECs under normal and hypoxic conditions and in a variety of disease states.

## EC Metabolism

Endothelial cells line the inner walls of arteries, capillaries, veins, and the lymphatic system, where they support tissue growth and repair by regulating the supply of nutrients and oxygen throughout the body. ECs are largely quiescent in healthy adults under normal physiological conditions but are activated by angiogenic signaling mechanisms in response to injury or pathological conditions (De Bock et al., 2013). Cytokines such as fibroblast and vascular-endothelial growth factor (FGF and VEGF, respectively) bind to their cognate receptors on ECs, which promotes vessel growth by guiding the proliferation of stalk ECs and the migration of tip ECs (Jakobsson et al., 2010; Falkenberg et al., 2019). EC function is also regulated by oxygen levels in the bloodstream (Giaccia et al., 2004; Semenza, 2014) via a number of oxygen-sensitive molecules, such as NADPH oxidases, endothelial nitric oxide synthase (eNOS), and heme oxygenase (Fraisl et al., 2009), as well as a specialized class of oxygen sensors, the prolyl hydroxylases (PHDs), which are crucially involved in the regulation of cell metabolism (Aragones et al., 2009).

**FIGURE 1:**
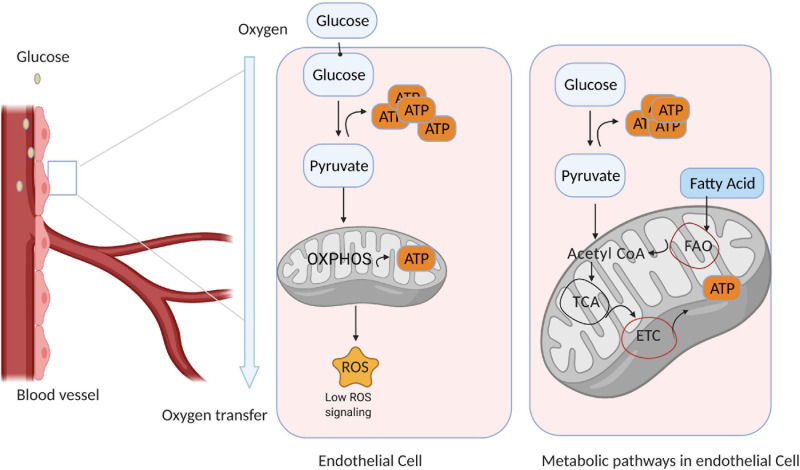
Endothelial cell (EC) metabolism is dominated by glycolysis. Quiescent ECs line the blood vessels and generate most of their ATP via glycolysis; however, a small amount of ATP is generated via mitochondrial metabolism, which also produces reactive oxygen species (ROS) that participate in signal transduction. Thus, ECs consume only a small amount of oxygen, and the remainder is available to support the activity of perivascular cells. (*Right*) ECs use glycolysis as the primary source of ATP generation, while other metabolic pathways are used in different conditions and often context dependent.

The mitochondrial content of ECs is quite low, comprising just 2–3% of total cytoplasmic volume (Falkenberg et al., 2019). Thus, most ECs depend on glycolysis for up to 85% of ATP production (De Bock et al., 2013), even in oxygen sufficient conditions (Li et al., 2019b; Figure 1). In fact, the amount of glucose consumed via glycolysis in ECs rivals that in cancer cells (Vander Heiden and DeBerardinis, 2017; Wong et al., 2017; Li et al., 2019a), and because the demand for oxygen in ECs is low, more is available to support the activity of perivascular cells. Anaerobic metabolism also reduces the production of ROS and enables ECs to not only resist hypoxic damage (provided glucose remains plentiful) (Mertens et al., 1990) but also to vascularize oxygen-deficient tissues. In quiescent ECs, glycolytic gene expression is regulated by the transcription factor FOXO1, which also suppresses endothelial growth and proliferation (Wilhelm et al., 2016). However, vascular endothelial growth factor (VEGF) upregulates glycolytic flux in ECs by both increasing the expression of glucose transporter 1 (GLUT1) (Yeh et al., 2008) and 6−phosphofructo−2−kinase/fructose−2,6−biphosphatase-3 (PFKFB3), which subsequently activates the rate-limiting glycolytic enzyme phosphofructokinase−1 (PFK−1) (Batori et al., 2020). Thus, PFKFB3 inhibition reduces glycolytic flux in ECs by approximately 35% (De Bock et al., 2013), leading to a corresponding decline in EC proliferation and migration (Xu et al., 2014), whereas PFKFB3 overexpression accelerates glycolytic flux and vessel sprouting (De Bock et al., 2013).

Intermediates of the glycolytic pathway can also be used as substrates for ATP production via mechanisms such as the hexosamine biosynthesis and pentose–phosphate pathways (HBP and PPP, respectively). The HBP pathway uses glucose, glutamine, acetyl-CoA, and uridine to generate N-acetylglucosamine (Wong et al., 2017) while also regulating the glycosylation and activity of proteins such as VEGFR-2 (Chandler et al., 2017), whereas PPP contributes to two different oxidative pathways: reversible and irreversible oxidative PPP. Reversible PPP is rate limited by transketolase (TKT) and generates ribose-5 phosphate, which is used for nucleotide biosynthesis (Bierhansl et al., 2017), whereas irreversible oxidative PPP is limited by glucose-6-phosphate dehydrogenase (G6PD) and produces both NADPH (Stanton, 2012), which is used for fatty acid synthesis, and NADH, which maintains redox homeostasis by functioning as a cofactor for endothelial nitric oxide synthase (eNOS) (Eelen et al., 2015). Notably, PPP inhibition reduces the viability and migration of ECs (Stanton, 2012; Falkenberg et al., 2019), while pulmonary arterial hypertrophy (PAH) is accompanied by an increase in PPP flux, which likely supports EC hyperproliferation by providing the substrates for DNA replication (Fessel et al., 2012; Cantelmo et al., 2016).

A number of other metabolic pathways, including fatty acid oxidation, glutamine and asparagine metabolism, and serine metabolism are also active in ECs. Particularly, ECs can directly uptake most of the fatty acids (FAs) from the circulated blood to fuel the catabolic processes and esterify excess FAs to form lipid droplets for storage (Eelen et al., 2018). Additionally, ECs can synthesize FAs endogenously by catalyzing acetyl-CoA to manoyl-CoA 2. However, these processes can lead to excessive intracellular FA accumulation, which impairs insulin signaling and glucose uptake and cause insulin resistance (Ghosh et al., 2017). Therefore, the elevation of EC-regulated lipids and free fatty acids in the circulated blood could be considered a valuable prognosticator for EC dysfunctions in several diseases such as diabetes and obesity. Ultimately, targeting the signaling molecules involved in fatty-acid-mediated EC dysfunctions could pave the way toward developing potential therapeutic approaches against EC dysfunction-related diseases.

ECs also consume a large portion of glutamine from the circulated blood to make protein and nucleotides for biosynthesis. In addition, ECs use glutamine as a major carbon source for the TCA cycle in order to survive. To illustrate, in cultured ECs, glutamine deprivation prevents ECs proliferation (Kim et al., 2017). For biosynthesis of other amino acids, glutamine is essential because it serves as a nitrogen donor for asparagine biosynthesis in ECs to sustain cellular homeostasis (Falkenberg et al., 2019). Asparagine then coordinates the metabolites TCA cycle intermediates for biosynthesis of these non-essential amino acids (Zhang et al., 2014). Collectively, regulating glutamine and asparagine may provide opportunities for antiangiogenetic diseases therapeutically. Furthermore, ECs can take up serine from the extracellular milieu or synthesize it from glycolytic intermediate 3-phosphoglycerate via serine synthesis pathway (SSP). Moreover, ECs depend on SSP for heme synthesis (Vandekeere et al., 2018). However, if heme accumulates in an excessive manner in endothelial cells, this can impair angiogenesis by triggering paraptosis.

## HIF and the Hypoxia Response Pathway

The HIF protein is a heterodimer composed of α and β subunits (HIFα and HIFβ, respectively), both of which belong to the basic helix–loop–helix, Per/Arnt/Sim (bHLH-PAS) superfamily of transcription factors; the α subunit is inducible and oxygen sensitive, whereas the β subunit is constitutively active and functions as an aryl hydrocarbon receptor nuclear translocator (ARNT) (Kewley et al., 2004; Bartoszewski et al., 2019). Three HIFα isoforms have been identified in mammals (HIF1α, HIF2α, and HIF3α) (Graham and Presnell, 2017), each of which is encoded by a distinct gene (Dayan et al., 2008; Wu and Rastinejad, 2017; Drevytska et al., 2018). HIF1α and HIF2α both contain a nuclear localization signal (NLS) motif and two transactivating domains, one located near the C-terminus (CTAD) and one near the N-terminus (NTAD) (Wu et al., 2015), whereas HIF3α lacks the CTAD (Hara et al., 2001; Hu et al., 2007). HIF1α and HIF1β are ubiquitously expressed in most cell types, while HIF2α and HIF3α expression is generally limited to vascular ECs, type II pneumocytes, renal interstitial cells, liver parenchymal cells, and cells of the myeloid lineage (Loboda et al., 2010; Majmundar et al., 2010). We have previously demonstrated that ARNT/HIFβ expression is highest in the heart (Wu et al., 2014). HIF3α messenger RNA (mRNA) is also abundant in tissues from the human heart, placenta, and skeletal muscle, but its function is not well-characterized (Maynard et al., 2003).

**FIGURE 2:**
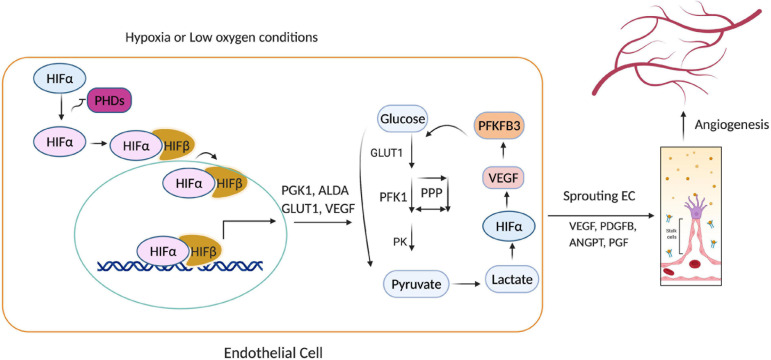
Hypoxia-inducible factor 1α (HIF1α)-mediated endothelial cell (EC) angiogenesis. Under hypoxia, prolyl hydroxylases (PHD1-3) cannot catalyze the hydroxylation of HIFα subunits, so HIFα avoids ubiquitin-mediated proteasomal degradation and translocates into the nucleus, where it activates genes that regulate glycolysis (PGK1, GLUT1, ALDA) and angiogenesis (VEGF, PDGFB, ANGPT). Thus, low oxygen availability causes HIF1α to accumulate, which promotes glycolytic flux, EC proliferation, and the sprouting of new capillaries.

HIF activity is primarily regulated by prolyl hydroxylases (PHDs) (Kaelin and Ratcliffe, 2008), which belong to a superfamily of dioxygenases that require iron(II) and 2-oxoglutarate (2-OG), as well as molecular oxygen and ascorbate, for their catalytic activity (Aragones et al., 2009; Pugh and Ratcliffe, 2017). In oxygen-sufficient conditions, PHDs promote the ubiquitin-mediated proteasomal degradation of HIF by marking (i.e., hydroxylating) specific proline residues on the HIFα subunit for ubiquitination via the von Hippel–Lindau (pVHL) E3 ubiquitin ligase complex (Bruick and McKnight, 2001; Maxwell et al., 2001). Thus, when molecular oxygen is scarce, HIFα accumulates in the cytosol and is translocated (via its NLS) into the nucleus, where it dimerizes with HIFβ, and the HIFα/β dimer binds to hypoxia-response elements (HREs) in the promoter of target genes, thereby regulating the expression of proteins that mediate the cellular response to hypoxia, and promote EC angiogenesis (Figure 2; Lee et al., 2004; Wei and Yu, 2007; Bartoszewski et al., 2019). In preconditioned myocardium, HIF1α is stabilized both in the nucleus and cytoplasm. HIF-1α into the nucleus through the nuclear pore complex is regulated by nuclear transport receptors (Depping et al., 2008). As long as PHDs are inactive, HIF turnover does not depend on the DNA binding, but some HIF target genes can induce degradation of HIF (Eckle et al., 2008). Notably, although HIF stability is primarily regulated by oxygen, HIF is also stabilized via interactions with c-Jun activation domain-binding protein-1 (Jab) (Shackleford and Claret, 2010) as well as the ROS produced by ionizing radiation, environmental stress, and the activity of angiopoietin-2 (Cash et al., 2007; Krock et al., 2011). Moreover, PHD regulation mechanism is also known to occur for other stress-inducible factors, such as activating transcription factor 4, an oxidative stress-inducible transcription factor (Moulin et al., 2020).

**FIGURE 3:**
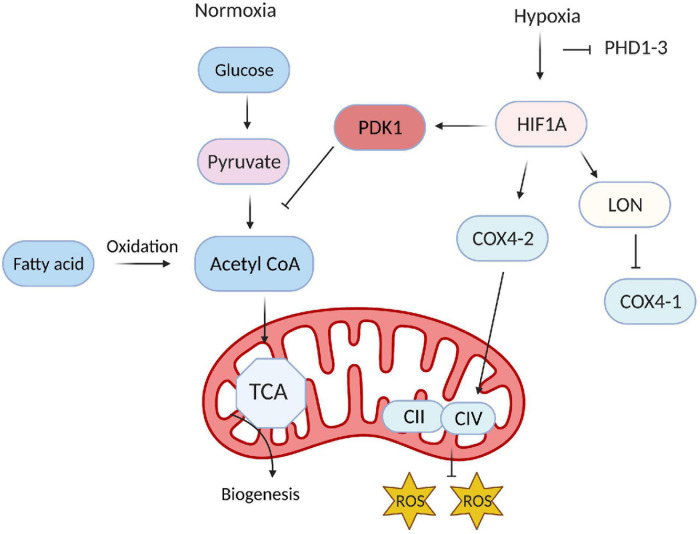
Hypoxia-inducible factor (HIF) reinforces glycolytic metabolism in endothelial cells (ECs). Under normoxic conditions, only a small proportion of the products of glycolysis in ECs are converted into acetyl CoA and enter the TCA cycle; instead, most of the acetyl-CoA is generated via fatty-acid metabolism and used for biomass production. During hypoxia, HIF reinforces the dependence of ECs on glycolysis by inducing the expression of PDK1, which prevents the conversion of pyruvate to acetyl-CoA.

As HIF accumulates in response to hypoxia, it induces the expression of many glycolysis-related genes, such as phosphoglycerate kinase (PGK-1), aldolase A (ALDA), and GLUT1 (Maltepe et al., 1997). HIF also upregulates pyruvate dehydrogenase kinase (PDK), which then phosphorylates pyruvate dehydrogenase (Holness and Sugden, 2003; Kim et al., 2006; Gordan et al., 2007; Okamoto et al., 2017) and blocks the conversion of pyruvate to acetyl-CoA. Thus, since acetyl-CoA is the primary substrate of the tricarboxylic acid cycle (TCA), HIF reinforces the dependence of ECs on glycolytic metabolism (Figure 3) by inhibiting TCA-induced OXPHOS. HIF1α and HIF2α also regulate the activity of the last component of the mitochondrial electron transport chain, cytochrome c oxidase (COX), by inducing a swap of subunit 4 (COX4) isoforms: HIF1/2α upregulates COX4-2 expression while simultaneously inducing the expression of LON, which degrades COX4-1 (Fukuda et al., 2007). The metabolites of glycolysis and the TCA cycle can also stabilize HIF by antagonizing 2-OG (Lu et al., 2005) and PHDs (Isaacs et al., 2005; Selak et al., 2005).

## HIF-Mediated Regulation of EC Metabolism in Vascular Disorders

### Myocardial Ischemia

Myocardial ischemia occurs when the demand for oxygen in the heart muscle exceeds the available supply, often because an intracoronary thrombus has disrupted blood flow to the heart (Koeppen et al., 2018). The resulting hypoxia upregulates both HIF1α (Lee et al., 2000) and HIF2α (Wiesener et al., 2003) in cardiac ECs, which increases ischemic tolerance (Eltzschig and Eckle, 2011) and triggers vessel growth by stimulating the expression of numerous angiogenic factors, including angiopoietins (ANGPT1 and ANGPT2) and VEGF (Pugh and Ratcliffe, 2003; Manalo et al., 2005). Angiopoietins are required for remodeling the vascular plexus, while VEGF induces EC proliferation (Yancopoulos et al., 2000; Semenza, 2003) and glycolytic flux (Fraisl et al., 2009; De Bock et al., 2013), which facilitates the sprouting of new capillaries (Shweiki et al., 1992; Liu et al., 1995). HIFs are also crucially involved in embryonic vascularization: HIF1α knockout mice die with vascular abnormalities on embryonic day 11 (E11) (Ryan et al., 1998), and genetic inactivation of HIFβ causes defects in angiogenesis of the yolk sac (as well as branchial arch formation) with embryonic lethality at E10.5 (Maltepe et al., 1997). Notably, the loss of HIF1α in ECs inhibits hypoxia-induced VEGF expression, indicating that HIF1α and HIF2α are not redundant and that HIF2α cannot compensate for the loss of HIF1α (Tang et al., 2004; Skuli and Simon, 2009). The role of EC ARNT/HIFβ expression in ischemia cardiac vascular diseases is not known and needs to be further investigated.

When blood flow is restored after prolonged ischemia, ROS production and inflammation increase, which can lead to ischemia–reperfusion injury (IRI) (Wu et al., 2018). IRI is primarily driven by apoptosis-induced cell death and is more harmful to ECs than to cardiomyocytes; thus, endothelial dysfunction is commonly observed in patients recovering from myocardial ischemia and can be associated with both morbidity and mortality (Shimokawa and Yasuda, 2008). IRI induces tissue damage via a variety of mechanisms, including a decline in cellular cAMP levels accompanied by increases in vascular permeability and leakage (Ogawa et al., 1992) as well as systemic increases in ROS production, declines in NO bioavailability, and imbalances in Ca^2+^ (Singhal et al., 2010). Notably, HIF1α also induces NO-dependent cardioprotection under normoxic conditions by inactivating PHD2, which increases NO production (Natarajan et al., 2006), and some evidence suggests that ischemic preconditioning protects the myocardium from subsequent IRI by stabilizing HIF (Eckle et al., 2008): monocyte-specific HIF2α deletions are associated with infarct expansion during IRI, whereas the induction of HIF2α by epithelial growth factor amphiregulin (AREG) reduces IRI-induced myocardial damage (Koeppen et al., 2018).

### Atherosclerosis

Atherosclerosis is most commonly associated with symptoms such as angina pectoris, heart attack, stroke, and claudication (Kannel et al., 2008; Frostegard, 2013), which are caused by perturbations in arterial blood flow (Rodrigues et al., 1997; Souilhol et al., 2020). The expansion of atherosclerotic lesions appears to proceed via plaque angiogenesis: a network of capillaries grows from the adventitial vasa vasorum into the intimal layer of the lesion (De Bock et al., 2013; Wong et al., 2017), leading to the accumulation of nutrients, lipids, and inflammatory cells in the arterial wall (Camare et al., 2017). The high oxygen demand of inflammatory cells, combined with the thickening of the intima (which impedes oxygen diffusion), reduces the local oxygen supply, thereby promoting neovascularization (Carmeliet, 2003; Rey and Semenza, 2010) and activating HIF. Thus, both HIF1α and HIF2α accumulate during atherosclerosis, and increases in HIF abundance are associated with the progression from early to late-stage lesions (Sluimer et al., 2008). Furthermore, the EC-specific deletion of HIF1α reduced lesion formation 6 weeks after arterial injury in mice fed a high-fat diet (Akhtar et al., 2015). HIF overexpression increased lesion size, and HIF inhibition reduced both VEGF activity and neointimal hyperplasia (Christoph et al., 2014), in atherosclerosis-prone (ApoE^–/–^) mice, and both genetic and pharmacological (FG4497) PHD inhibition reduced plaque surface area by ∼50% in mice lacking the receptor for low-density lipoprotein (Rahtu-Korpela et al., 2016). EC HIF1α activity also promotes the recruitment of atherogenic monocytes (depending on the availability of miR-19a) (Akhtar et al., 2015), and Krüppel−like factor 2 (KLF2), which suppresses glycolytic flux in normal ECs by downregulating PFKFB3, is itself downregulated during atherosclerosis, which may partially explain why hypoxia increases glucose uptake in atherosclerotic plaques, especially in macrophage-rich areas (Folco et al., 2011).

### Pulmonary Hypertension

Pulmonary hypertension (PH) is characterized by the remodeling of small pulmonary vessels and a progressive increase in pulmonary vascular resistance. Thus, both hypoxia, which promotes vascular remodeling and vasoconstriction (Stenmark et al., 2006), and the HIF transcriptional system are crucially involved in the development of PH. HIF1α/β is expressed in medial smooth muscle cells of the pulmonary artery, as well as in the endothelial plexiform lesions associated with PH (Tuder et al., 2001; Fijalkowska et al., 2010), and pulmonary response is delayed in heterozygous HIF1α-deficient mice, whereas HIF2α heterozygous mice are resistant to PH and right-ventricular dysfunction (Brusselmans et al., 2003). PH is also characterized by an increase in the proliferation of pulmonary arterial endothelial cells (PAECs), and arachidonate 5-Lipoxygenase (ALOX5) is one of the key metabolites responsible for EC proliferation and pulmonary vasoconstriction: when exposed to hypoxia, the ALOX5 pathway is upregulated in human PAECs, which increases H_2_O_2_ production and, consequently, H_2_O_2_-dependent EC proliferation (Porter et al., 2014). Whether HIF participates in the hypoxia-induced activation of ALOX signaling in ECs has yet to be thoroughly investigated; however, the ALOX5 promoter contains putative binding sites for both early growth response protein 1 (EGR1) and specificity protein 1 (SP1), which function with HIF as coregulators of erythropoietin receptor expression in lung cancer cells (Su et al., 2019). Notably, glucose uptake is significantly upregulated in both the lungs and ECs of patients with idiopathic PAH (IPAH) and is accompanied by declines in EC mitochondrial density and higher rates of EC proliferation (Tuder et al., 2001; Fijalkowska et al., 2010; Cao et al., 2019), while heterozygous PFKFB3 deficiencies and the administration of a PFKFB3 inhibitor protected mice and rats, respectively, from hypoxia-induced PH (Cao et al., 2019). Thus, the role of HIF in EC metabolism may also contribute to the development or progression of PH. Therefore, HIF-mediated EC pathways and their downstream targets could be novel therapeutic options to PH.

### Diabetic Endothelial Dysfunction

Elevated blood glucose levels in patients with diabetes can significantly alter EC metabolism and lead to endothelial dysfunction (Knapp et al., 2019). High glucose levels activate protein kinase C (PKC) and increase both nitric oxide synthase (NOS) and superoxide production in cultured ECs; however, some evidence suggests that PKC-mediated phosphorylation of NOS reduces NO production (Hink et al., 2001), and declines in NO bioavailability coupled with increases in oxidative stress contribute to vascular dysfunction in diabetes. Insulin resistance in patients with type 2 diabetes (T2D) may also be an independent contributor to vascular dysfunction, as evidenced by observations that endothelium-dependent vasodilation is impaired in patients with insulin resistance who are otherwise healthy, as well as in animal models of insulin resistance (Wheatcroft et al., 2003).

The hypoxic regulation of glucose uptake (Airley and Mobasheri, 2007) is at least partially mediated by the HIF-induced upregulation of VEGF, which subsequently promotes GLUT expression. Thus, a number of studies have shown that HIF function is disrupted in patients with diabetes; for example, both HIFα and VEGF expression was downregulated in heart-tissue samples and biopsies from T2D patients who underwent coronary bypass graft surgery (Maltepe et al., 1997). The downregulation of HIF1α appears to be attributable to an increase in fatty-acid metabolism because fatty-acid elevations reduce the availability of succinate, which is required for HIF1α accumulation (Dodd et al., 2018). Therefore, a novel therapy aiming at stabilizing HIF1 proteins in diabetic hearts could target succinate. Moreover, our recent studies show a significant reduction in ARNT/HIF1β in cardiac microvascular endothelial cells isolated from diabetic animal models, suggesting an essential role for endothelial ARNT/HIF1β in the diabetic heart. However, the underlying mechanism in which ARNT/HIF1β is regulated in diabetes needs to be further investigated. Prolonged exposure to hyperglycemia also impairs endothelial function by downregulating endothelial NOS expression (Connell et al., 2007), which inhibits HIF1α accumulation (Gao et al., 2014), and by increasing the production of ROS and reactive nitrogen species (RNS) (Sena et al., 2013), which promote HIF1α degradation by activating PHDs.

## Conclusion

Vascular ECs maintain cardiovascular health by regulating the extravasation of nutrients and signaling molecules from the blood, by producing factors that regulate tone and other properties of the vasculature, and by participating in mechanisms that are crucial for recovery from injury. Notably, their role in tissue repair is facilitated, at least in part, by their dependence on glycolysis for ATP generation, which enables them to resist hypoxic damage and promote angiogenesis in ischemic regions. The physiological response to hypoxia is largely regulated by HIF, and, consequently, HIF activity can either attenuate or exacerbate the progression and severity of many, if not all, diseases that are associated with cardiovascular complications and endothelial dysfunction. Thus, continued investigations of the mechanisms that regulate—and are regulated by—HIF in specific organs, tissues, and disease states will facilitate the development and refinement of treatments for a wide variety of cardiovascular disorders.

## Author Contributions

KU: writing-original draft. RW: writing, supervision, reviewing, and editing. Both authors contributed to the article and approved the submitted version.

## Conflict of Interest

The authors declare that the research was conducted in the absence of any commercial or financial relationships that could be construed as a potential conflict of interest.
